# Evaluation of diet pattern related to the symptoms of mexican patients with Ulcerative Colitis (UC): through the validity of a questionnaire

**DOI:** 10.1186/s12937-015-0014-3

**Published:** 2015-03-14

**Authors:** Nallely Bueno-Hernández, Martha Núñez-Aldana, Ilse Ascaño-Gutierrez, Jesús K Yamamoto-Furusho

**Affiliations:** 1 Inflammatory Bowel Disease Clinic, Department of Gastroenterology, Institute of Medical Sciences and Nutrition “Salvador Zubirán”, Vasco de Quiroga No. 15, Col. Sección XVI, Del, Tlalpan CP 14000 Mexico City, Mexico; 2 Postgraduate Unit, National Autonomous University of Mexico (UNAM), Circuito de Posgrado S/N, Del, Coyoacán, Ciudad Universitaria, CP 04510 Mexico City, Mexico

**Keywords:** Ulcerative Colitis, Diet, Exacerbation of symptoms, Relapse

## Abstract

**Background:**

Ulcerative Colitis (UC) is a chronic disease characterized by inflammation of colonic mucosa. Environmental factors such as dietary patterns may increase symptoms in UC patients.

**Aim:**

To validate and implement a questionnaire to identify foods that exacerbates symptoms in UC patients.

**Methods:**

A prospective cohort study was conducted to validate and to assess the relationship of food and symptoms in Mexican UC patients.

**Results:**

The IVC obtained was 0.56 in the questionnaire and kappa = 0.03 in foods from animal origin, 0.5 cereals and tubers, 0.2 legumes, 0.4 vegetables and fruits, 0.4 fats and 0.3 in others. The evaluation was carried out in UC patients (n = 233), 65% active and 35% in UC remission, the current age was 45 (SD; 15) years in active UC and 40 (SD; 15) years in UC remission. Three food groups were made based on the frequency of symptoms: Group A; symptoms more often, especially the active vs remission (P <0.05); Group B caused more symptoms in remission UC vs active UC (*P* = 0.07) and Group C caused more symptoms in the active UC (*P* = 0.05).

**Conclusion:**

Foods with higher frequency of symptoms in patients with UC were: beans, whole milk, plum, lima beans and spicy sauce.

## Background

Ulcerative Colitis (UC) is a chronic condition characterized by inflammation of the colonic mucosa and belongs to a subgroup of the Inflammatory Bowel Disease (IBD) [1].

Epidemiological studies have shown a significant increase in the incidence of UC around the world (approximately 3 million of people), most frequently in Asian and Western countries [1,2]. In Mexico, a study reported an increased incidence of UC from 28 to 78 cases in the period from 1987 to 2006, mainly in adults between 21 and 30 years [3].

The etiology of UC remains unknown, however, environmental factors such as diet, modulate the immune response to bacterial components in individuals with genetic susceptibility; diet patterns have emerged in order to improve the activity of UC. In European countries, an increase in the incidence of IBD has been associated with a gradual change of diet, by consuming large quantities of vegetables, fruits and fish to a pattern of westernized diet, with larger amount of processed meat, refined grains, foods high in fats, sweet foods and drinks with high sugar levels, which could perpetuate the disease activity through changes in microbiota particularly by waste metabolites [2,4-6]. In the United States of America (USA) the incidence of IBD has been increased and associated with high intake of simple carbohydrates in recent years [6,7].

On the other hand, the diet during the periods of activity plays an important role to exacerbate the symptoms. Cohen et al. published a study in which food such as vegetables, nuts and fried foods, milk, red meat, soft drinks, popcorn, dairy products, alcohol, spicy foods, fruits, foods rich in fiber and fat, corn, seeds, beans and coffee aggravate symptoms of patients with IBD, even a diet with high consumption of sweetened beverages, cheese, pizza, milk and processed meat associated with increased risk to the presence of stomas in patients with CD, but foods such as yogurt, rice and bananas produced a decrease in the frequency of symptoms [8]. In Canada another study showed through a questionnaire, that other foods such as: chocolate, bran cereal, soft drinks, Mexican food, foie gras and artificial sweeteners cause discomfort in patients with IBD, without determining the type of symptom and specific food [9].

Diet patterns can exacerbate symptoms in patients with UC, however, the kind of food that exacerbates the symptoms in Mexican patients have not been documented accurately, and if these changes are influencing in the disease activity.

The aim of this study was to validate and apply a questionnaire determining the foods that may increase the symptoms in patients with UC.

## Methods

A prospective cohort study was performed for the validation of the questionnaire and a cross-sectional study, to evaluate food related to the symptoms of the disease in patients with definitive diagnosis of UC confirmed by histopathology; the study was approved by the committee of research and ethics in our Hospital.

### Validity of the questionnaire

The questionnaire to evaluate symptoms caused by each food was validated according to 2 criteria: content and construct. Content validity is the degree to which an instrument has an appropriate sample of items for the construct being measured and is an important procedure in the scale development. Content validity index (CVI) is the most widely used index in quantitative evaluation, which through a group of experts evaluates the usefulness of items describing each one as unnecessary, useful and necessary. The construct validity, refers to the degree to which two measures of constructs that theoretically should be related, are in fact related and can be estimated using correlation coefficients like the test Cohen Kappa, to validate the questionnaire in the second step, a random sample was taken from patients with UC to who was applied the baseline questionnaire and 3 months later was applied the same questionnaire in order to assess the concordance of their responses in two different periods of time under the same clinical scenario (active or remission).

### Questionnaire design

Each questionnaire evaluated 6 food groups: 1) Foods from animal origin, 2) Cereals and Tubers, 3) Legumes, 4) Vegetables and Fruits, 5) Oils and fats, and 6) Others (foods of low nutritional value); as well as also the relationship of these with the most common symptoms of UC: diarrhea, bloating, abdominal pain and flatulence, in order that each patient identified one symptom that every food caused him to consume it. Clinical data, endoscopic, histological and biochemical activity of the disease were also evaluated.

### Study population for the questionnaire application

Patients were selected by consecutive sampling in the Inflammatory Bowel Disease Clinic at the National Institute of Medical Sciences and Nutrition in the period between 2012 and 2014. The sample size was obtained considering a value of alpha (α) of 5%, 95% confidence level and 1.96 of value of z, with the following equation n = (0.25)(N)/(α/z)2(N-1) + 0.25, we obtained at least 185 patients with UC but finally more patients were recluted with a total of 233 patients. The sample was selected according to the residence of the patients (North, Centre and South of Mexico) to ensure a representative sample of all the eating habits in the whole country.

### Statistical analysis

For the validity of the content of the questionnaire was applied the test of CVI, which considered reference values that range from - 1 to 1 and it is considered an ideal value for the sample of 0.51. For the validity analysis, the index of concordance of two 2 questionnaires applied to patients in the period of 3 months, for this test Cohen Kappa (kappa) which considered reference values are used ranging from 0 to 1, where was taken as acceptable a value greater than 0.3.

The description of the results was done with means and standard deviations. For the analysis of the results by group applied the test of Kolmogórov-Smirnov in order to evaluate the distribution of the data and determine the type of statistics to use for which was determined that the data did not have a normal distribution. To determine if there was a difference in the frequency of symptoms (present or absent) for each food test, X^2^ and compare the average frequency of symptoms among the active vs remission and spread of disease, by each food group, the U Mann Whitney test was used. The data was analyzed with the statistical packages SPSS version 17 and Med Calc. A P value < 0.05 was considered as significant.

## Results and discussion

### Validity of the questionnaire

In this study, we validated a questionnaire that allowed to identify the frequency of the most common symptoms in patients with UC caused by the consumption of different kind of foods from whole country.

For the CVI, 14 experts were consulted, 7 nutritionists and 7 gastroenterologists who had experience in the treatment of patients with UC, each surrendered a questionnaire in order to rate the utility of including each of the food groups proposed, with the symptoms of UC mentioned in the instrument (diarrhea, rectal bleeding, bloating, abdominal pain and flatulence), on this assessment, the CVI by food group and symptoms of all the instrument was 0.56 (min. 0.14 and Max 0.86).

For the construct validity analysis, a prospective cohort study were evaluated 132 patients, 60% were men, with an average of 41 (SD; 15) years age, 73% had active UC and 77% had pancolitis. The foods evaluated were divided previously into 6 groups and the concordance index (kappa) was obtained of each questionnaire at the beginning and after 3 months for each of the groups of food by symptom, the kappa value was 0.3 for foods from animal origin, 0.5 for cereals and tubers, 0.2 for legumes, 0.4 for vegetables and fruits, 0.4 for oils and fats and 0.3 for others (foods of low nutritional value) as shown in Table 1.

**Table 1:** **Kappa index by food group evaluated in the validity of the questionnaire and foods included in each group**

	Kappa	IC 95%
**Food of animal origin**	0.3	0.25 - 0.35
Tunny, barbecue, red meat, fried meat, pork, sausages, ham or sausage, egg, seafood, fish, chicken or turkey, gut or belly, skim milk, lactose-free milk, whole milk, fresh cheese, cured cheeses, sour cream, drinking yogurt and solid yogurt.		
**Cereals and tubers**	0.5	0.48 - 0.58
Rice, oats, sugar cereal, fiber cereal, sugar cookies, salted cookies, granola, white bread, sweet bread, brown bread, potato, pasta soup, flour tortillas, corn tortillas.		
**Legumes**	0.2	0.11 - 0.26
Beans, lima beans, lentils and textured soy.		
**Vegetables and fruits**	0.4	0.36 - 0.42
Chard, beetroot, broccoli, pumpkins, cauliflower, green beans, spinach, tomato, lettuce, nopales, cucumber, chutney, ketchup, vegetable soup, plums, strawberry, guava, tangerine, mango, apple, melon, orange, papaya, raisins, pear, pineapple, banana, watermelon, tuna and grapes.		
**Oils and fats**	0.4	0.24 - 0.49
Vegetable oil, butter, butter and oilseeds.		
**Others**	0.3	0.30 - 0.45
Alcohol, chocolates, coffee, cigars, French fries, ice, industrialized juices, jams and soda.		

### Association between food and symptoms

For the application of the questionnaire and evaluation of symptoms by food, we evaluated a total of 233 patients with UC, 20% were from North part, 17% South and 63% from the Centre of Mexico (Table 2). Of the 233 patients included in this phase of the study, 65% (n = 151) had active UC and 35% (n = 82) were under remission, with age average of 45 (SD; 15) years and 40 (SD; 15) years respectively, 77 (51%) patients with active UC and 49 (60%) patients who had remission were male, the time evolution average was 5 years, 86 patients had active UC and 29 patients with UC in remission had pancolitis.

**Table 2:** **Epidemiological characteristics from 233 UC patients**

	Remission	Active
	n = 82	n = 151
Age	40(±15)	45 (±15)
Sex (m/f)	32/49	74/77
Evolution time (years)	10	8
Extension (Distal/Pancolitis)	11/29	44/86
Presence of extraintestinal manifestations (%)	21	46
Intolerance to food groups		
Dairy	38	62
Spicy foods	19	36
Fat	12	25
Vegetables and fruits	13	10

**Figure 1:**
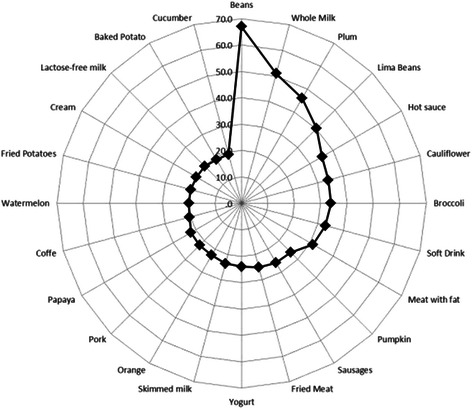
**This chart graphically details the 24 foods with increased frequency of symptoms in patients with UC: beans 67%, whole milk 51%, plum 46%, lima beans 40%, chutney 35%, cauliflower 34%, broccoli 34%, soft drink 33%, red meat with fat 31%, pumpkin 26%, sausage 26%, fried meat 25% , yogurt 24%, skim milk 24%, orange 23%, pork 26%, papaya 22%, coffee 20%, watermelon 20%, French fries 20%, cream 20%, lactose-free milk 20%, baked potato 19% and cucumber with 19%.**

**Figure 2:**
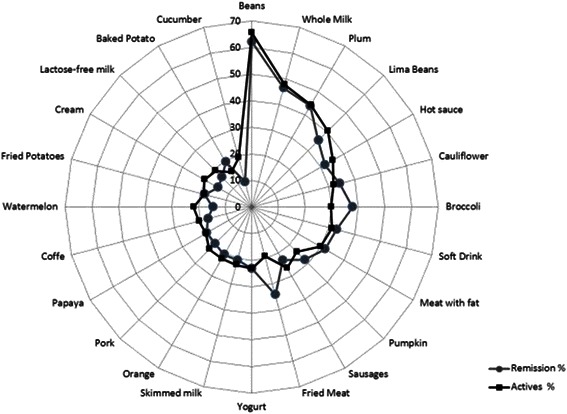
**Food distribution with increased frequency of symptoms in active (black line) and remission UC patients (gray line).** Patients in remission had increased frequency of symptoms with foods such as broccoli, soft drink, meat with fat, pumpkin, fried meat and baked potato, compared to patients with active.

Of the 6 food groups included in the questionnaire, 81 foods were evaluated, and those that caused increased frequency of symptoms (24 foods) were dairy products, legumes and crucifers (Figure 1). The difference in symptoms between assets and remission was evaluated with each one of the foods with increased frequency of symptoms and found that the behavior of symptoms differed significantly in some foods (Figure 2).

**Figure 3:**
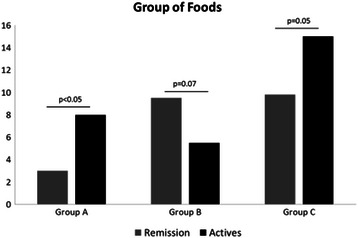
**Frequency of symptoms in the Group A or high, B or medium and C or low in active and remission UC patients.**

Three groups of food were clustered according to the frequency of symptoms: Group A or high, Group B or medium and Group C or low. The foods from Group A (beans, whole milk, plum, lima beans and Chutney) resulted in increased frequency of symptoms in all patients, especially those with active UC compared to patients with UC in remission (Group A; P < 0.05); in the Group B (cauliflower, broccoli, soda, red meat with fat, pumpkin, sausage and fried meat) found that patients in remission had significantly increased frequency of symptoms compared to patients with active UC (Group B; P = 0.07) and Group C (yogurt, skim milk, orange, pork, papaya, coffee, watermelon, French fries, cream, lactose-free milk, baked potato and cucumber) foods that cause symptoms in both groups but more often in patients with activity (Group C; P = 0.05) as shown in Figure 3 and Table 3. These findings showed that there was a close relationship between the frequency of symptoms, and certain type of foods from the daily diet such as: dairy, legumes and crucifers, these are similar findings in Canada, where determined in a general manner that the Mexican food resulting in increased frequency of symptoms [9], in this study we were able to assess the type of specific food that causes symptoms in Mexican patients with UC.

**Table 3:** **Food groups with increased frequency of symptoms divided by active and remission status**

Group	Food	Remission n = 82 (%)	Actives n = 151 (%)	P
**A (High)**	Beans	62	66	<0.05
	Whole Milk	46	48	
	Plum	44	44	
	Lima Beans	35	40	
	Chutney	32	35	
**B (Medium)**	Cauliflower	34	32	0.07
	Broccoli	38	30	
	Soda	33	31	
	Meat with fat	32	30	
	Pumpkin	28	24	
	Sausages	23	26	
	Fried Meat	34	19	
**C (Low)**	Yogurt	23	23	0.05
	Skim milk	21	23	
	Orange	21	23	
	Pork	20	23	
	Papaya	20	20	
	Coffee	17	21	
	Watermelon	15	22	
	French fries	18	19	
	Cream	15	21	
	Lactose-free milk	16	19	
	Baked Potato	20	15	
	Cucumber	10	19	

**Figure 4:**
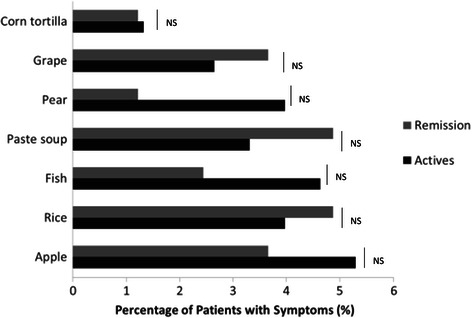
**Foods not associated with symptoms in active and remission UC patients.**

We observed that there are several foods containing high simple carbohydrates and saturated fatty acids (FA) in the groups of food (A and B), this finding is accordingly to the study reported by Brown et al. who described that the excessive intake of carbohydrates and saturated FA causes imbalance of microbiota [10] causing imbalance in the Th1 and Th17 response as well as inhibition of the interleukin 10 (IL-10) production and increasing inflammation [11-13]. Furthermore, we evaluated foods not associated with symptoms (less than 6%) (Figure 4) and our results demonstrated that foods rich in omega-3 (vegetable oils) no produced symptoms in UC patients. This effect can be explained because omega-3 (EPA and DHA) reduces the production of IL-6 and IL-8 through the activated Peroxisome Proliferator-Activated Receptor (PPARγ) in the enterocyte and has been associated with a decrease of 77% of the severity of the symptoms; and even combined with 5-Aminosalicylates (5-ASA) improves the inflammatory response and mild clinical course of disease [14-18].

Another group of foods that caused symptoms in patients with UC were the cruciferous and dairy in Group C (yogurt, skimmed milk, orange, papaya, watermelon, coffee, fried potato chips, cream, lactose-free mil, baked potato and cucumber). This effect may be related to the findings from Laing et al. who showed that genetic polymorphisms in DIO1 (Hormonal Regulation Peroxidase) and HLA (which regulate the activity of the IL1 and IL6) causes poor absorption of crucifers, overpopulation of E. Coli and increased inflammation in IBD patients [19]. Dairy products tend to result in a rate of sensitivity in patients with IBD ranging from 10 to 20% especially in those patients with inflammation in the terminal ileum. The findings of the present study found that sensitivity to dairy was 41% in active UC patients and 47% in remission with UC. This intolerance can be explained by enzymatic deficiencies secondary to inflammation producing a deficient absorption of lactose, fat and protein from the milk [20,21]^.^

**Figure 5:**
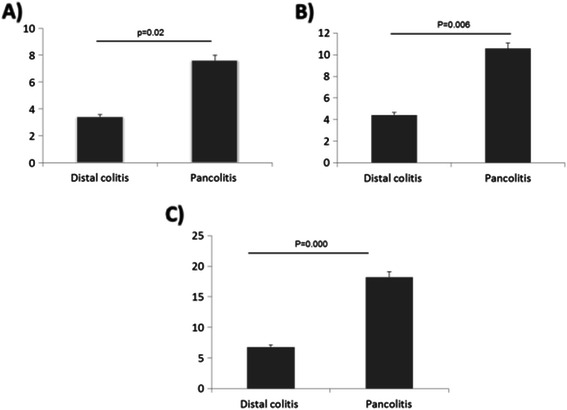
**Frequency of symptoms to food from Group A (Figure5A), Group B (Figure5B) and Group C (Figure5C) according to extent of disease in UC patients.** Pancolitis had significant increased frequency of food intolerance.

The groups with increased frequency of symptoms (Groups A, B, and C), were evaluated if the spread of the disease could determine any difference in the frequency of the symptoms and found that there is increased significant frequency of symptoms in patients with pancolitis compared to those who had distal colitis, being this statistically significant difference (P = 0.02).(Figure 5).

Therefore, restricted pattern of diets have been suggested in patients with UC, for example, partially free oligosaccharides, disaccharides and monosaccharides as well as Fermentable Polyols (FODMAPs) from refined flours, vegetables, fruits. Some studies have suggested that can decrease the episodes of diarrhea in patients with IBD [22,23]; In this study, some foods from groups A and B belonged to the FODMAPs such as beans, milk, soda, lima beans, cauliflower and pumpkin. Thus, exclude these foods can help relieve UC symptoms; similarly, anti-inflammatory diets with changed in the dairy products, refined or processed sugar, lean meats, vegetables, fruits and nuts, reduce the frequency of symptoms [24]. We found that the restrictions of these diets are consistent, especially considering significant differences in the activity of UC and the extent of disease *(P* < 0.05) due to make excessive restrictions in patients with UC may exacerbate deficiencies several nutrients such as: calcium, iron, vitamin B12, vitamin D and folic acid.

## Conclusion

The present study validated a tool for evaluating the food intake and its association with symptoms in patients with UC. Several foods can cause increased frequency of symptoms in UC patients such as beans, whole milk, plum, lima beans and spicy sauce. On the other hand, there is a subset of foods that cause symptoms in patients who are in remission.

## Abbreviations

(UC): Ulcerative Colitis
(IBD): Inflammatory Bowel Disease
(CD): Crohn’s Disease
(CVI): Content validity index
(kappa): Cohen Kappa
(FA): Saturated Fatty Acids
(IL-10): Interleukin 10
(PPARγ): Peroxisome Proliferator-Activated Receptor
DIO1: (Hormonal Regulation Peroxidase)
(FODMAPs): Fermentable Oligosaccharides, Disaccharides and Monosaccharides Polyols
